# Role of TAM Receptors in Antimalarial Humoral Immune Response

**DOI:** 10.3390/pathogens13040298

**Published:** 2024-04-02

**Authors:** Lijo John, Rahul Vijay

**Affiliations:** 1 Department of Veterinary Biochemistry, Kerala Veterinary and Animal Sciences University, Pookode 673576, Kerala, India; 2 Center for Cancer Cell Biology, Immunology and Infection, Rosalind Franklin University of Medicine and Science, North Chicago, IL 60047, USA; 3 Department of Microbiology and Immunology, Rosalind Franklin University of Medicine and Science, North Chicago, IL 60047, USA

**Keywords:** phosphatidylserine, malaria, plasmablasts, hypoxia, AXL, *Plasmodium*, humoral immune response, sterilizing immunity

## Abstract

Immune response against malaria and the clearance of *Plasmodium* parasite relies on germinal-center-derived B cell responses that are temporally and histologically layered. Despite a well-orchestrated germinal center response, anti-*Plasmodium* immune response seldom offers sterilizing immunity. Recent studies report that certain pathophysiological features of malaria such as extensive hemolysis, hypoxia as well as the extrafollicular accumulation of short-lived plasmablasts may contribute to this suboptimal immune response. In this review, we summarize some of those studies and attempt to connect certain host intrinsic features in response to the malarial disease and the resultant gaps in the immune response.

## 1. Introduction

Malaria, transmitted by infected female *Anopheles* mosquitoes, is caused by an apicomplexan parasite, *Plasmodium* spp. The major symptoms of malaria range from fever, chills, to severe anemia, sometimes resulting in debilitating neurological symptoms to even death [1]. The disease is widespread in tropical and subtropical regions of the world, predominantly in Sub-Saharan Africa. Disease severity varies due to the differences in the geographical distribution of different strains of *Plasmodium* and the genetic peculiarities of the host population [2]. Despite decades of investigation, malaria remains a major global public health threat with an estimated 249 million cases and 608,000 related deaths in 2022 [3]. The burden of the disease is further exacerbated by *Plasmodium*-immune evasion strategies, continuing emergence of drug-resistant strains, and suboptimal humoral immune memory, resulting in repeat infections [4]. Persistent public health measures and interventions to eliminate malaria were showing promise (with incidence rates dropping nearly 18%) over the last decade; however, the COVID-19 pandemic redirected the focus of the scientific and medical community, reversing these gains at least partially.

A successful immune response should provide adequate protection from an ongoing infection and safeguard the host from repeat infections. Identifying molecules and mechanisms underlying the inefficient induction of humoral immune memory and investigating ways to rectify them using immunotherapeutic approaches is vital to reducing the malaria burden. As a part of the Malaria Vaccine Implementation Program, in October 2021, the World Health Organization recommended the roll out of the first ever malaria vaccine RTS, S/AS01, in 18 countries of the sub-Saharan region where malaria is endemic. To further complement this vaccination approach, a second malaria vaccine called R21/Matrix-M was added to the program in October 2023. It remains to be seen how these vaccines will be able to protect against repeat infections and whether the elusive sterile immunity against *Plasmodium* can be effectively generated.

Our major goal with this review is to bring to attention some cellular and molecular characteristics intrinsic to the pathophysiology of malaria that may be critical to the development of suboptimal effector and memory immune response following *Plasmodium* infection. We especially focus on the role of a membrane phospholipid, phosphatidylserine (PtS), that becomes exposed on the outer leaflet of the RBC membrane during malaria-induced hemolysis and how it may regulate anti-*Plasmodium* immune response.

## 2. Pathophysiology of Malaria

**Figure 1:**
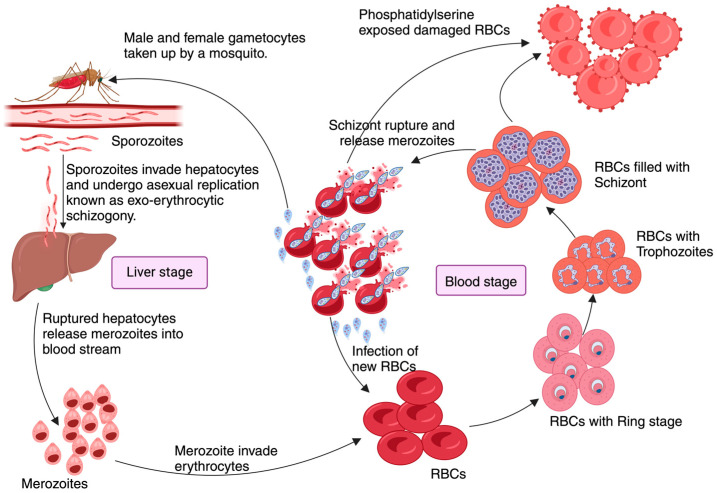
Pathophysiology of Malaria: *Plasmodium* pathogenesis begins when an infected female *Anopheles* mosquito transfers motile sporozoites during a blood meal. These sporozoites invade hepatocytes after a brief resting period at the injection site initiating the ‘Liver stage’ of infection. Here, they undergo several cycles of asexual reproduction known as exo-erythrocytic schizogony, which usually takes approximately 9–16 days, depending on the *Plasmodium* species. Infected hepatocytes release merozoites into the blood which will invade the RBCs, initiating the ‘Blood stage’ of infection. Here, merozoites transform into various intra-erythrocytic forms such as the ring stage, trophozoite, and schizonts. The rupture of RBCs loaded with schizonts releases cellular content, parasites, and metabolic products into circulation, triggering immune reactions. The merozoites that are then released from the schizonts re-infect RBCs, perpetuating the blood stage of infection. Meanwhile, male and female gametocytes, that will be taken by another *Anopheles* mosquito seeking a blood meal, will fuse to form zygotes in the gut of the mosquito, to later develop into ocysts, completing the cycle when ingested by a female *Anopheles* mosquito during another blood meal.

*Plasmodium* is one of the most complex eukaryotic pathogens completing its heteroxenous life cycle between a free-flying, blood-feeding dipteran vector and a vertebrate host [5]. Within the infected vector, the infectious forms of the parasite (sporozoites) reside in the salivary glands. During a blood meal, the motile sporozoites in the saliva gets injected into the skin of the primary host and rapidly migrate to the liver, infecting and multiplying in hepatocytes (Figure 1). This initiates the asymptomatic ‘liver stage’ of infection. Subsequently, invasive merozoites that are released from the hepatocytes proceed to infect red blood cells (RBCs), where they undergo repeated rounds of maturation, proliferation, egress, and invasion, initiating the ‘blood stage’ of the disease. This repeated cycle of the erythrocytic or blood stage of infection results in extensive hemolysis and anemia and is also the stage when most clinical symptoms of malaria are manifested.

Development of *Plasmodium* within the RBCs is marked by high demand for cholesterol and membrane phospholipids [6]. Parasites’ demand for membrane components leads to alterations in the RBC membrane, which is characterized by cholesterol depletion and modifications to membrane lipid composition [7,8]. The release of parasites and the parasite-induced membrane changes together contribute to the increased exposure of PtS on the outer membrane of the RBCs (Figure 1). In this review, we cite some of our ex vivo and in vivo studies using PtS blocking antibodies as well as bone marrow chimeras, showing that PtS exposed on the RBC membrane serves as an important immune modulator during *Plasmodium* infection [9]. We also discuss some of our published studies that show that detection and binding of PtS to certain receptors, predominantly the AXL receptor (detailed below) on B cells, may be critical to the proliferation and accumulation of a certain class of B cells called plasmablasts; these cells exercise an immunosuppressive role and may be key to the suboptimal immune response observed during malaria. Our future studies are aimed at elucidating the precise mechanism by which the pathophysiology of malarial disease regulates this novel pathway of plasmablast differentiation.

## 3. Anti-Malarial Immune Response

The immune response against malaria is complex and not yet completely understood. Among the different stages of infection, the liver stage is clinically silent compared to the blood stage of infection [10]. However, the liver stage of infection induces a strong innate immune response that is relevant for early control of parasite load and delaying progression to the blood stage. This response in the liver is dependent on interferon γ, type I interferon receptors, and natural killer T cells [11]. Unlike as in viral or bacterial infections, the type I interferon response elicited by malarial parasites has been shown to be independent of toll-like receptors or its downstream signaling, instead mediated by melanoma differentiation-associated gene 5 protein (MDA5) and mitochondrial antiviral signaling (MAVS) protein. The resulting interferon response was necessary for the recruitment of lymphoid cells to the liver [10,12]. Furthermore, studies also show that a fraction of the parasites injected into the skin following a mosquito bite are taken by CD11c+ dendritic cells in the draining lymph node, which eventually prime a potent CD8 T cell response against the sporozoite antigens [13]. These data show that although the liver stage shows minimal symptoms during malaria, it is far from immunologically inert.

**Figure 2:**
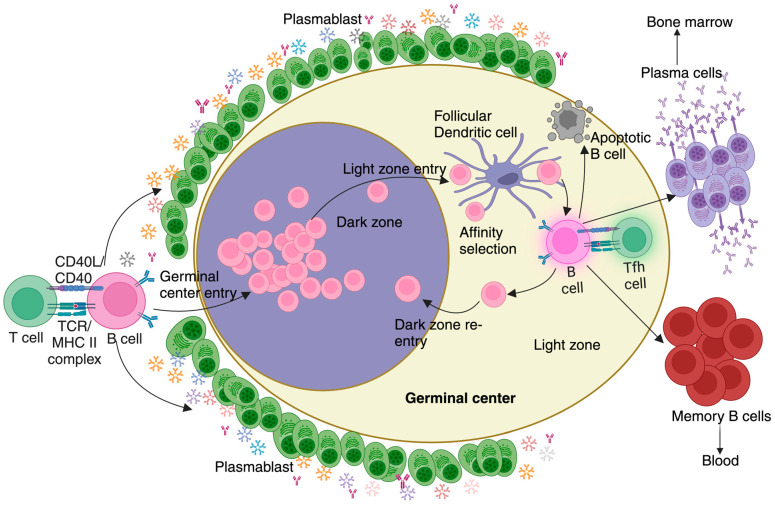
**Germinal center reaction during Malaria**: An effective T-dependent B cell response involves the antigen-exposed activated B cells undergoing a series of maturation steps processes known as the germinal center reaction. This reaction occurs within specialized areas of B cell follicles known as germinal centers, which consist of distinct dark and light zones. Following entry into the germinal center, B cells progress through several stages, including somatic hypermutation, affinity selection, and maturation. B cells that successfully navigate these selection processes differentiate into plasma cells, responsible for producing pathogen-specific, high-affinity antibodies and residing primarily in the bone marrow, and memory B cells, which rapidly differentiate into plasma cells upon re-exposure to the same antigen and are released into and retained in circulation. Before the initiation of the germinal center reaction, a small population of rapidly proliferating B cells secreting predominantly IgM, known as plasmablasts, appear outside the B cell follicle. In malaria, these hypermetabolic plasmablasts are disproportionately numerous. The high metabolic demands of plasmablasts successfully compete for key nutrients, effectively acting as a nutrient sink to deprive GC B cells from sourcing them. This metabolic constraint ultimately contributes to a suboptimal immune response against malaria.

The clinical signs observed during the blood stage of infection result from the release of inflammatory cytokines that follows a strong immune B-cell-centric adaptive immune response against *Plasmodium* [14]. Studies conducted in rodent malaria models, immuno-epidemiological studies, and even control human malaria infection studies have conclusively shown the importance of antibodies in protection from malaria [15,16,17]. Clearance of *Plasmodium* relies on spatially and temporally layered CD4 T-cell-dependent B cell activation events culminating in the production of parasite-specific antibodies [18,19,20]. As a blood-borne infection, immune activation takes place in and around distinct splenic regions called B cell follicles, which transform into more specialized structures called germinal centers (GC). The clearance of and protection from *Plasmodium* relies on GC-derived plasma cells (PC), which migrate to the bone marrow (BM) and secrete high-affinity parasite-specific antibodies, and memory B cells (MBCs) that remain in the spleen and circulation to later differentiate swiftly into PCs upon pathogen re-encounter [21]. However, several studies have shown that induction of MBCs following malaria is rather suboptimal and short-lived and even after being exposed to parasitic loads known to be sufficient to induce B cell responses [22,23]. Prior to the appearance of GC-derived PCs or MBCs, another class of B cells with a pronounced endoplasmic reticular (ER) network and Golgi complex accumulated outside the GCs [24] (Figure 2). These extrafollicular B cells called plasmablasts (PB) are short-lived and were believed to secrete low-affinity antibodies against *Plasmodium*, to essentially ‘*buy the host time*’ before the GC-derived high-affinity B cell response. Contrarily, our studies in rodent malaria models show that [24] these cells are in fact immunosuppressive and that their depletion enhanced humoral immune response, parasite control, and protection from reinfection (a sign of heightened MBC recall) [24]. Our studies also indicate that the immunosuppressive effect of PB is reversed by administering L-glutamine in drinking water to *Plasmodium*-infected mice. This was evidenced by enhanced parasite control, GC responses, and protection from lethal *Plasmodium* challenge along with heightened MBC-driven antibody responses in L-glutamine-administered mice [24]. Moreover, these effects of L-glutamine supplementation disappeared when PBs were depleted, showing that the immunosuppressive effects were indeed due to their (PB) functioning as a nutrient (L-glut) sink [24]. This paradigm shift on the role of early short-lived PB may have major implications, not only in malaria but also in other infections, such as COVID-19, Dengue, and Trypanosomiasis, in which accumulation of PB seems to not effectively control the pathogen or the disease [25,26,27,28]. In light of our published and unpublished observations, we discuss the putative role played by a family of receptor tyrosine kinases that comprises of Tyrosine protein kinase receptor 3(TYRO3), AXL and Membrane receptor tyrosine kinase (MERTK), together abbreviated as TAM (TYRO3, AXL, MERTK) in governing the outcome of GC responses during *Plasmodium* infection.

## 4. TAM Receptor Biology

**Figure 3:**
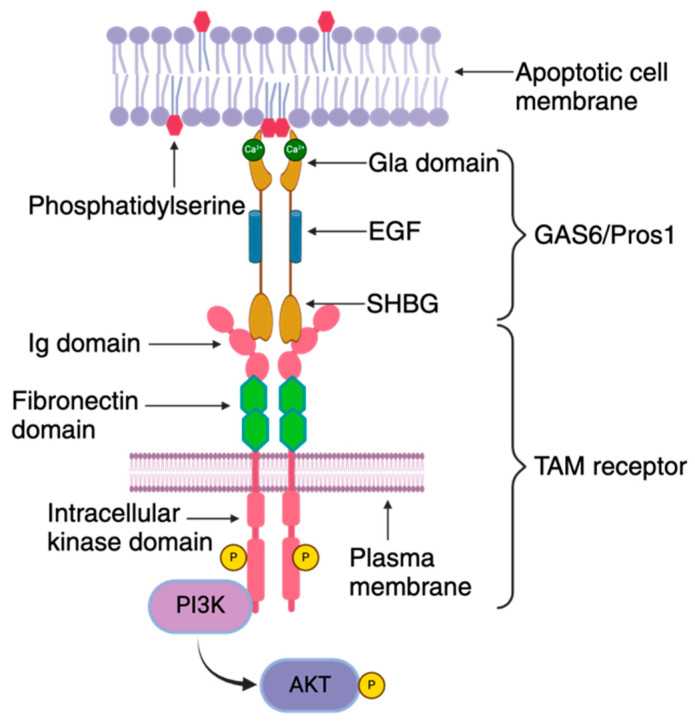
**TAM receptor organization**: The TAM receptor is comprised of distinct structural elements, including an extracellular domain, a transmembrane segment, and an intracellular kinase domain. The extracellular region features an immunoglobulin domain responsible for binding to its ligands GAS6/PROS1, followed by tandem fibronectin type III domains. This is followed by a single-pass transmembrane domain and an intracellular protein tyrosine kinase domain. The TAM ligands, GAS6/PROS1, consist of a Gla domain, an EGF domain, and a sex hormone-binding globulin (SHBG) domain. The Gla domain is notable for its γ-carboxylated glutamic acid residues, which enables binding to exposed phosphatidylserine on the outer leaflet of the RBC membrane in a Ca^2+^- and vitamin K-dependent manner. The SHBG domain contains two laminin G domains that bind to the immunoglobulin domain of the TAM receptor. These domains are separated by four epidermal growth factor (EGF)-related domains. Interaction between GAS6/PROS1 and the TAM receptor occurs in a 1:1 stoichiometry, leading to receptor dimerization and subsequent activation of the intracellular protein tyrosine kinase. This activation is coupled to the activation of the phosphoinositide 3 kinase (PI3K)/AKT pathway.

The TAM receptor tyrosine kinase family plays an important role in the phagocytosis of apoptotic cells, innate immune response, autoimmunity, spermatogenesis, and a plethora of other biological functions [29,30,31]. Unlike many other receptors, TAM receptors utilize serum factors such as Protein-S (PROS1) and growth arrest-specific-6 (GAS-6), as bridging molecules to bind to their ligands [32,33] (Figure 3).

Protein-S (PROS1) and growth arrest-specific-6 (GAS-6) are vitamin K-dependent soluble proteins, sharing 43% of the amino acid identity among them. PROS1 was originally identified as a cofactor for Protein C, which negatively regulates blood coagulation by degrading clotting factors Va and VIIIa [34]. PROS1 is capable of activating TYRO3 and MERTK but not AXL [32,35,36,37,38,39]. GAS6, on the other hand, while capable of binding to all three receptors (TAM), binds to AXL with the highest affinity [40]. Among the multiple families of PtS-binding proteins, the most studied are those with a Gla (γ-carboxylated glutamic acid residues) domain [41,42,43]. Both PROS1 and GAS6 are Gla domain-containing proteins which are capable of binding to negatively charged phospholipid groups in a calcium- and vitamin K-dependent manner [44,45,46]. The ability of PROS1/GAS6 to activate TAM receptors depends on the carboxylation of the Gla domain and its binding to phosphatidylserine (PtS) [47]. Apart from the Gla domain, the SHBG domain (composed of two laminin G domains) and four EGF-related domains are also part of the receptor complex. The SHBG domain is positioned at the carboxy-terminal end, and the EGF-related domain is present in between the SHBG and Gla domains [30,32,48] (Figure 3).

Expression of TAM receptors vary based on the cell type, even within the same lineage. For example, bone marrow-derived dendritic cells show higher expression of AXL than MERTK, whereas the bone marrow-derived macrophage population shows the opposite trend [49]. Both cell populations show very low expression of TYRO3. The expression of AXL was controlled at the translational level, but MERTK was found to be regulated transcriptionally [49]. Intriguingly, AXL and GAS6 exhibit a peculiar relationship, where AXL specifically needs GAS6 for its activation, while GAS6 relies on AXL for its stable maintenance, showing a co-dependency between the receptor and its ligand [49].

All receptor tyrosine kinases except insulin receptors exist as monomers in the cell membrane [50]. Upon ligand binding, RTKs are dimerized and activated, although each receptor employs different mechanisms of receptor dimerization [51,52,53]. The extracellular amino-terminal regions comprising of an Ig-like domain of TAM proteins bind with the laminin G domain of SHBG in PROS1/GAS6, followed by tandem repeats of fibronectin-type repeats, a transmembrane domain, and an intracellular tyrosine kinase domain [54,55,56,57,58,59]. TAM ligand binding leads to receptor dimerization in two steps, with a high-affinity GAS6/TAM complex forming first and autophosphorylation of the intracellular kinase domain [55,60]. The TAM receptor, upon binding to their ligand, has been shown to both homo- or heterodimerize to initiate signaling [61,62]. AXL exerts its effects by signaling via PI3K, MAPKs, and EGFR/PKC/mTOR pathways [63,64], depending on the nature of dimerization. Heterodimerization is reported when one of the TAM receptors is overexpressed. When TYRO3 is overexpressed, AXL signaling shifts from its predominant PI3K pathway to the MAPK pathway [61], indicating that the dimerization pattern of TAM receptors affects the outcome of GAS6-mediated signaling.

Phosphatidylserine (PtS) is a phospholipid normally restricted to the inner and cytoplasm-facing leaflets of the plasma membrane [65]. The caspase-mediated cleavage of flippases, a P4-type ATPase that restricts PtS to the inner leaflet, and the activation of a Ca^2+^-dependent enzyme scramblase lead to redistribution of PtS to the outer leaflet. The presence of PtS on the outer leaflet of the plasma membrane acts as an ‘eat me’ signal for the phagocytic immune cells [41,66,67,68]. Phagocytosis of the apoptotic cells that expose PtS on their outer membrane is carried out by MERTK-mediated signaling, which is elicited by either GAS6 or PROS1. Inflammatory signals promote PtS–GAS6-mediated phosphorylation of AXL and enhancement of AXL-mediated phagocytosis. This demonstrates that AXL and MERTK participate in phagocytosis under varying conditions, as they respond to different stimuli by interacting with different ligands [49]. This suggests that pathogens utilize the AXL–GAS6 pathway to gain entry into cells and/or even hide from the host immune response preventing their clearance [24,69,70,71,72,73,74,75].

## 5. TAM Receptors and Infections

### 5.1. AXL and Viral Infections

Several studies focusing on viral infections have documented that the immunosuppressive role of AXL occurs mainly through inhibition of the interferon-stimulating genes (ISG) [31,71,76,77]. Many enveloped viruses utilize PtS on their envelope to exercise apoptotic mimicry (‘eat me signal’) to attach to the host GAS6 molecule and thus AXL to elicit a signaling cascade facilitating AXL-mediated cell entry and inhibition of ISGs. This interference in the natural anti-viral response disrupts the innate immune response, facilitating the establishment of infection [78]. This hijacking of the GAS6–AXL signaling axis is reported in Ebola virus [79], Zika virus [71], Respiratory Syncytial Virus (RSV) [80], SARS-CoV-2 [74], and Parvovirus [75].

Region-specific deletion/inhibition studies in AXL revealed that the intracellular kinase domain is essential for the internalization of the virus [69,81]. While the presence or absence of TAM receptors (especially AXL) did not seem to affect Zika virus cell entry [70], AXL seems to attenuate the Zika virus-induced activation of type I interferon genes, thereby promoting virus infection [71]. This was confirmed by gene knock-out studies involving AXL and IFNαR [72]. In contrast, experiments in human glioblastoma cell lines using CRISPR-Cas-mediated knockdown of AXL showed its requirement for Zika virus cell entry and infection [73]. Thus, the role of AXL receptor appears to be different depending on the cell types they are expressed on.

Recent reports also suggest that AXL may act as a receptor to facilitate SARS-CoV-2 virus entry into pulmonary and bronchial epithelial cells. Interestingly, SARS-CoV-2 binding to AXL is independent of GAS6 or the virus envelope-associated PtS [74]. Human parvovirus B19 also shows similarities in AXL utilization, where AXL acts as a co-receptor for infection in human erythroid progenitor cells [75].

AXL appears to also have an indirect effect during RSV infection. RSV infection drives increased production of GAS6, leading to heightened AXL stimulation. This, in turn, impairs antibacterial immunity by attenuating caspase 1 activation and IL-18 release, rendering patients vulnerable to secondary pneumococcal infections. Elevated GAS6 levels also cause polarization of M0 to M2 instead to an M1 phenotype, further attenuating the antibacterial activity [82]. On the other hand, the absence of AXL promotes IL-1α from pyroptotic macrophages, compromising the integrity of the blood–brain barrier and permitting the neural invasion of Japanese encephalitis virus (JEV) [83]. Contrary to studies showing an inhibitory effect of AXL on ISGs, some studies have shown that AXL enhances interferon-stimulated genes, allowing for increased vulnerability to JEV infection of neurons [84], further reiterating the cell-specific role of AXL in regulating the immune response.

These contrasting findings in various infectious disease settings not only highlight the diverse roles played by AXL in modulating immune response and disease outcomes but also emphasize the importance of further investigating this seemingly ubiquitous PtS–AXL–GAS6 signaling axis.

### 5.2. AXL and Malaria

The role of AXL in malaria is poorly understood and investigated. *Plasmodium* parasites are not known to utilize TAM receptors to gain entry into the host cells like viruses do (discussed in Section 5.1). The only report connecting AXL and malaria stems from our studies showing that AXL ligation and signaling may be key to the accumulation of immunosuppressive pre-GC plasmablasts [9].

As mentioned earlier (Section 3), plasmablasts induced by *Plasmodium* infection express high levels of amino acid transporter molecules and act as a glutamine sink [24]. Our studies also revealed that plasmablast accumulation may require polyclonal activation signals, but it is largely independent of antigen-specific mechanisms and may still rely on certain pathophysiological features related to hemolysis. In fact, hemolysis-mediated exposure of PtS on RBCs during malaria has been previously reported [85]. Furthermore, the observation that *Plasmodium*-induced plasmablasts preferentially secrete anti-PtS antibodies led us to speculate that PtS may be involved in the differentiation of these cells. Importantly, our RNA sequencing studies revealed that resting and activated B cells that appear before the accumulation of plasmablasts expressed higher levels of many PtS receptors including AXL. Further studies using ex vivo stimulation approaches and in vivo blocking studies revealed that by binding to PtS, AXL may play a pivotal role in the expansion of polyclonal plasmablast [9] during *Plasmodium* infection. The direct cell-to-cell contacts required between PtS-exposed RBCs and AXL-expressing B cells for driving plasmablasts differentiation would also mean that plasmablasts may be derived from B cells located in the marginal zone rather than from the splenic white pulp due to their close proximity to one another. After all, marginal zone B cells localized in the red pulp have been shown to interact with RBCs and are considered important for antibody responses against blood-borne pathogens [86]. It is also important to note that, while B cell activation, differentiation, and proliferation preferentially happen in the white pulp, foci of antibody-secreting B cells have been observed in the red pulp [87,88,89]. Whether these B cells are indeed short-lived plasmablasts or are germinal-center-derived needs to be validated.

Splenic red-pulp macrophages that have been shown to play a major role in RBC clearance may also act as a bridge between PtS-exposed RBCs and B cells that are destined to become plasmablasts. Expression of TAM receptors, especially AXL, has been documented in macrophages [33], although there is no evidence to suggest that this may regulate anti-*Plasmodium* humoral immune response. However, the gross disruption of splenic architecture as well as the formation of the blood–spleen barrier [89] early during *Plasmodium* infections likely prevents the interaction of red-pulp macrophages and RBCs. These notions regarding the role of marginal zone B cells as the precursors of polyclonal immunosuppressive plasmablasts or lack of role played by red-pulp macrophages in this phenomenon need to be further validated.

Nevertheless, most of our data indicate that accumulation of plasmablasts that are detrimental to an upcoming or ongoing germinal center response are unique to malaria and, to some extent, Trypanosoma infection [9]. However, what pathophysiological factors specific to malaria contribute to this phenomenon is still not clear. Hemolysis and systemic hypoxia develop as early as four days post infection with *Plasmodium* [90,91]; higher levels of HIF1α were also noticed in placental malaria cases [92]. Targeted metabolomics data of *Plasmodium*-infected mouse serum showed elevated orotate and lactate levels (*unpublished data*), a characteristic feature of systemic hypoxia [93]. Resting and activated B cells in the spleen of the *Plasmodium*-infected mice also showed a more hypoxic signature in comparison with plasmablasts, indicating that proliferation of plasmablasts is favored by a cellular hypoxic environment. Studies have also independently shown that alleviation of the hypoxic status increases the probability of survival in a mouse cerebral malaria model [94,95]. Understanding how systemic hypoxia promotes the progression of malaria is crucial in designing strategies to improve anti-malarial immunity.

**Figure 4:**
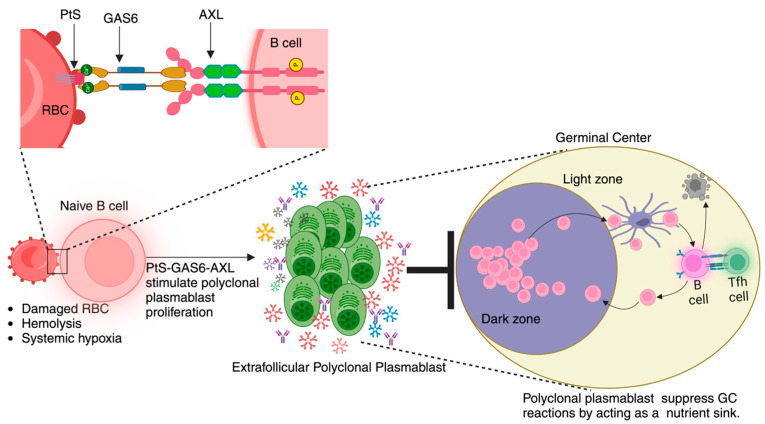
**Working model**: We present a theoretical framework outlining the processes that occur during the development of malaria. The model centers around the interactions between *Plasmodium*-triggered surface exposure of phosphatidylserine (PtS) on red blood cells (RBCs), hemolysis-driven hypoxic state, and AXL receptor expression on B cells. Upon binding, this PtS–GAS6–AXL signaling cascade promotes the proliferation of polyclonal plasmablast population. The heightened metabolic activity of plasmablasts induces a state of nutrient deprivation and creates a competitive environment for key nutrients, which, in turn, limits the availability of resources for germinal center cells. This nutrient deprivation compromises the efficiency of germinal center reactions, ultimately leading to a dampened immune response against the pathogen.

There have been several attempts to establish a molecular link between AXL and hypoxia-inducible factors. Studies have shown that hypoxia and hypoxia-inducible factor (HIF)1 can stabilize AXL expression and signaling [96,97] as well as support plasma cell differentiation [98,99]. HIF1 and HIF2 are also known to directly bind to the hypoxia response elements in the AXL promoter region and enhance its expression [97]. Furthermore, exogenous supplementation of GAS6 was shown to downregulate AXL protein expression without affecting the transcription of *Axl* mRNA in in vitro tumor environment [96]. This indicates a regulatory role of GAS6 over AXL at the translational level, although reports supporting this claim are few and far between. There are also reports that AXL increases HIF levels during hypoxia [100]. To further add complexity to the already sophisticated signaling axis, overexpression of AXL has been shown to increase translation of HIF1α without altering the mRNA levels, and this effect of AXL was dependent on PI3K/Akt/p70^S6K^ signaling cascade [101]. Thus, there are different levels of regulation of the AXL–GAS6–PtS signaling axis, and hypoxia appears to have a significant yet underappreciated role in his phenomenon. Given that malarial infection is characterized by (Figure 4) (i) the appearance of immunosuppressive plasmablasts, (ii) hemolytic events, (iii) systemic hypoxia, and (iv) PtS exposure, more mechanistic studies aimed at investigating these pathophysiological features may reveal critical new information to devise immunomodulatory therapies to enhance anti-malarial immunity.

## 6. Summary

Studies show that gradual acquisition of protective or clinical immunity is evident following multiple malarial episodes; however, sterilizing immunity is rarely achieved. This makes the older population in malaria endemic regions relatively asymptomatic during the disease, but still acting as critical reservoirs of the pathogen, facilitating inadvertent transmission. Among the many factors that may result in the suppression of an optimal anti-malarial immune response (so that sterilizing immunity is achieved), we identified that the pre-GC extrafollicular plasmablast may also contribute to this phenomenon. This population of B cells that appear early during *Plasmodium* infection is short-lived, yet metabolically hyperactive and seem to deprive the GC B cells from key nutrients, especially L-glutamine. Our recent studies also show that a key member of the TAM receptor tyrosine kinases, AXL, plays a significant role in the differentiation of these immunosuppressive cells by recognizing and binding to PtS exposed on the surface of RBCs. Hemolytic anemia and the ensuing hypoxia during malaria may likely stabilize AXL, thereby facilitating the cascade of events leading to the differentiation of plasmablasts. While the role of AXL in tumor progression, phagocytosis, cell proliferation, immune cell interactions, and autoimmunity is well studied, its role in B-cell-mediated immune response is not yet fully understood. Moreover, given that all three TAM receptors bind to similar ligand(s), it is possible that TYRO3 and MERTK may also have synergistic or redundant roles in modulating anti-malarial immune responses. Further mechanistic insight can be achieved by investigating their temporal and cell-specific role during infection. Such studies will not only aid in the development of specific inhibitors of these receptors (some are already in clinical trials) but will also help in exploring the possibility of using them in combinatorial therapeutics alongside anti-malarial ones.
